# Associations between Interleukin-32 Gene Polymorphisms rs12934561 and rs28372698 and Susceptibilities to Bladder Cancer and the Prognosis in Chinese Han Population

**DOI:** 10.1155/2020/8860445

**Published:** 2020-11-05

**Authors:** Jie Yang, Zhongyu Jian, Pengfei Shen, Yunjin Bai, Yin Tang, Jia Wang

**Affiliations:** ^1^Department of Urology, Institute of Urology, West China Hospital, Sichuan University, Chengdu, Sichuan 610041, China; ^2^Department of Urology, Chengdu First People's Hospital, Chengdu, Sichuan 610041, China

## Abstract

The proinflammatory chemokine interleukin-32 is related to various diseases, including cancer. However, it has never been associated with bladder cancer (BC). To detect whether there is a relationship between the *IL-32* gene polymorphisms (rs12934561 C/T and rs28372698 T/A) and BC, the study enrolled 170 non-muscle-invasive bladder cancer (NMIBC) patients, 151 muscle-invasive bladder cancer (MIBC) patients, and 437 healthy controls. The polymerase chain reaction-restriction fragment length polymorphism (PCR-RFLP) method was used for the *IL-32* single-nucleotide polymorphism (SNP) genotyping. Statistical analysis was performed using SNPstats online analysis software and SPSS software. Our data revealed that the CC homozygous genotype of rs12934561 in BC patients was significantly higher than that in controls (*P* = 0.03, OR = 1.47, 95%CI = 1.04‐2.08), and the percentage of TC genotype carriers was relatively less than that of controls (*P* = 0.001, OR = 0.61, 95%CI = 0.45‐0.82). Furthermore, the TT homozygous genotype of rs28372698 was associated with a significantly lower overall survival rate in MIBC patients (*P* = 0.028, OR = 2.77, 95%CI = 1.11‐6.90). The *IL-32* gene polymorphism rs12934561 might be associated with increased BC risk, and the rs28372698 might participate in the prognosis of BC patients. Therefore, they could be potential forecasting factors for the prognosis of MIBC patients.

## 1. Introduction

Bladder cancer (BC) is the tenth most common cancer according to the International Agency for Research on Cancer (IARC), with 549,393 new cases worldwide in 2018 (1). Seventy-five percent of the total burden occurs in men, and 60% of the incidence rate and 50% of the mortality rate occur in the less developed regions of the world. In 2018, 82,270 new cases and 38,208 deaths were recorded in China, which revealed an estimated increase of 30,000 cases and 20,000 deaths compared with the data of 2012 (1). According to these reports, only about 20% of BC patients have muscle-invasive bladder cancer (MIBC), which is responsible for most of the cancer-specific deaths. The remaining 80% of the patients present with non-muscle-invasive bladder cancer (NMIBC) (1–3).

Common BC risk factors include tobacco smoking and exposure to industrial paints, petroleum products, and other chemical carcinogens (2–4). However, in recent years, increasing evidence has demonstrated a genetic predisposition towards it (5). Furthermore, the first-degree relatives of BC patients have a twofold higher risk of developing BC, showing that genetic factors play a crucial role in the initiation and progression of this disease.

Interleukin-32, a proinflammatory cytokine, was first detected as the product of natural killer cell transcript 4 (NK4) in 1992 (6) and was officially renamed as IL-32 by Kim et al. in 2005 (7). Its encoding gene *IL-32* is located on the human chromosome 16p13.3, is approximately 1,200 bp full-length, and consists of eight exons (6). IL-32 is mainly produced by activated T cells, NK cells, epithelial cells, and blood monocytes (7), and it has nine splice variants IL-32*α*, IL-32*β*, IL-32*γ*, IL-32*δ*, IL-32*ε*, IL-32*θ*, IL-32*ζ*, IL-32*η*, and IL-32small (IL-32sm) (8, 9). IL-32 has been implicated in many inflammatory diseases and cancers, including rheumatoid arthritis (10), chronic obstructive pulmonary disease (COPD) (11), lymphoma (12), head and neck squamous cell carcinoma (HNSCC) (13), thyroid cancer (TC) (14), hepatocellular carcinoma (HCC) (15), lung cancer (LC) (16–18), esophageal cancer (19), gastric cancer (GC) (20, 21), pancreatic cancer (22), colorectal cancer (CRC) (23, 24), renal cell carcinoma (RCC) (25), breast cancer (26), and endometrial cancer (EC) (27).

Recently, several reports have clearly indicated that two single nucleotide polymorphisms (SNPs) in the *IL-32* gene sequence (rs12934561 and rs28372698) were associated with cancer susceptibility (LC, GC, TC, EC, and CRC) (14, 16, 24, 27–29). However, no relationship has been established between IL-32 and BC. Therefore, we selected these two SNPs (rs12934561 and rs28372698) of *IL-32* to determine their differences in BC patients and healthy controls in the Chinese Han population.

## 2. Material

### 2.1. Participants' Clinical Characteristics

A case-control study which enrolled 321 unrelated BC individuals (mean ± SD: 63.82 ± 12.17 years (NMIBC group: 62.14 ± 12.87 years; MIBC group: 65.70 ± 11.06 years)) and 437 healthy controls (mean ± SD: 63.86 ± 6.94 years) was approved by the hospital ethics committee, and informed consent was provided by all the participants. The subjects were from the West China Hospital of Sichuan University between 2007 and 2012. All participants with personal or family history of BC or other severe diseases such as other types of cancers, or those who had undergone radiotherapy or chemotherapy, were excluded from the study. Patients' clinical and follow-up data were collected every 6 months for 5 years by telephone calls. All tumor tissues resected from BC patients were confirmed by histopathological analysis, and the clinical characteristics are summarized in Table 1. All of the participants were genetically unrelated individuals of the Han population living in the Sichuan province of China.

### 2.2. Genotyping

As shown in Table 2, polymerase chain reaction (PCR) primers of the two SNPs were designed using Primer 3 web version 4.1.0. (http://primer3.ut.ee/) (30). The genetic DNA of each individual was extracted from a 200 *μ*L EDTA-anticoagulated peripheral blood sample using a DNA isolation kit from BioTeke (Peking, China). Genotyping was performed using PCR-restriction fragment length polymorphism (PCR-RFLP). The DNA fragments that contained the polymorphisms were amplified in a volume of 10 *μ*L, including 100 ng extracted genomic DNA, 2.7 picomole primers of each SNP, and 5 *μ*L 2x power Taq PCR Master Mix (BioTeke, Peking, China). The PCR annealing temperature was 60°C for 30 s. After PCR termination, the products were digested by a restriction enzyme, as shown in Table 2, and the digested fragments were separated on a 6% polyacrylamide gel and stained with 1.5 g/L of argent nitrate. Finally, DNA sequencing analysis was used to confirm the genotypes, and approximately 10% of the randomly selected samples were 100% in agreement with the results after performing the repeated assays.

### 2.3. Statistical Analysis

The SNPstats online analysis software was used to evaluate the genotypic association, including the codominant, dominant, recessive, and overdominant genetic models (31), and the Hardy-Weinberg equilibrium was calculated using the chi-squared test. The effects of different genotypes and alleles were evaluated by odds ratio (OR) and respective 95% confidence intervals (95% CI). Kaplan-Meier univariate analysis plots and Cox regression multivariate survival analysis model were used to estimate the relationships of *IL-32* genotypes with patient outcomes. The level of significance was set at *P* < 0.05.

## 3. Results

### 3.1. Susceptibility between the *IL-32* Genotypes and BC

The genotype distributions of these two SNPs follow the Hardy-Weinberg equilibrium (*P* > 0.05) in our groups. The effects of *IL-32* genotypes and allele frequencies on BC patients are presented in Table 3. As shown, for rs12934561, the homozygous genotype (CC) in the recessive genetic model was significantly higher in BC patients than that in controls (24.6% vs. 18.1%, *P* = 0.03, OR = 1.47, 95%CI = 1.04‐2.08), indicating an increased risk for BC susceptibility. Compared with the TT/CC genotypes, the TC genotype was associated with a lower risk for BC in the overdominant model (*P* = 0.001, OR = 0.61, 95%CI = 0.45‐0.82). No significant differences were observed between BC susceptibility and the rs28372698 genotype or allele distribution.

### 3.2. Clinical Characteristics

To gain further insights into the relationship between these two SNPs of *IL-32* and BC, patients with different genotypes were stratified by mean age (≤64 and >64 years old), sex (male and female), smoking status (smokers and nonsmokers), tumor grade (low-grade and high-grade), and tumor stage (Ta-T1 and T2-T4) (Supplementary Table 1). However, no significant relationship was detected for any subgroup of the SNPs after adjusting for common risk factors (*P* > 0.05).

### 3.3. The Effects of *IL-32* SNP Genotypes on Patient Outcome

During the follow-up, all of the involved BC patients were tracked every six months. At the end of our study, 50 patients (15.6%, NMIBC: 13 cases, MIBC: 37 cases) died of BC and 95 patients (29.6%, NMIBC: 48 cases, MIBC: 47 cases) relapsed. Following the stratification of patients by tumor stage (MIBC and NMIBC), we conducted Kaplan-Meier survival analyses and multivariate Cox survival analyses; the associations between SNPs of *IL-32* and BC patient outcomes are summarized in Table 4.

**Figure 1:**
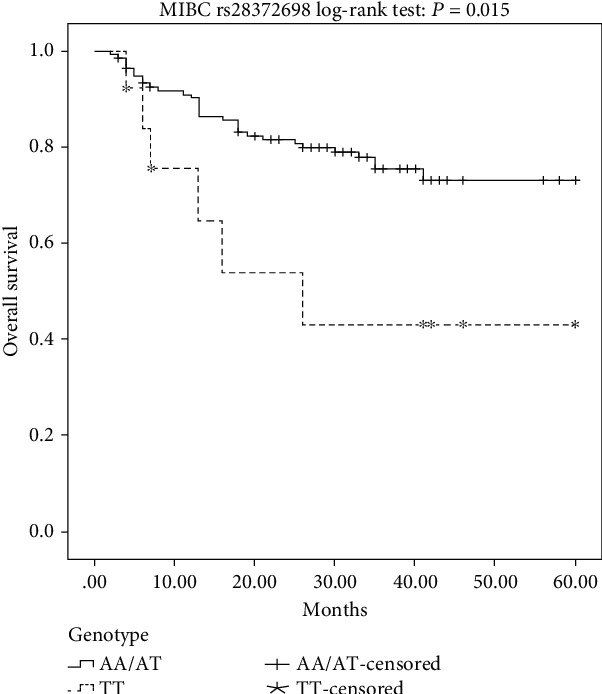
Kaplan-Meier overall survival curves for all of the analyzed MIBC patients categorized by *IL-32* rs28372698 in the recessive genetic models.

**Figure 2:**
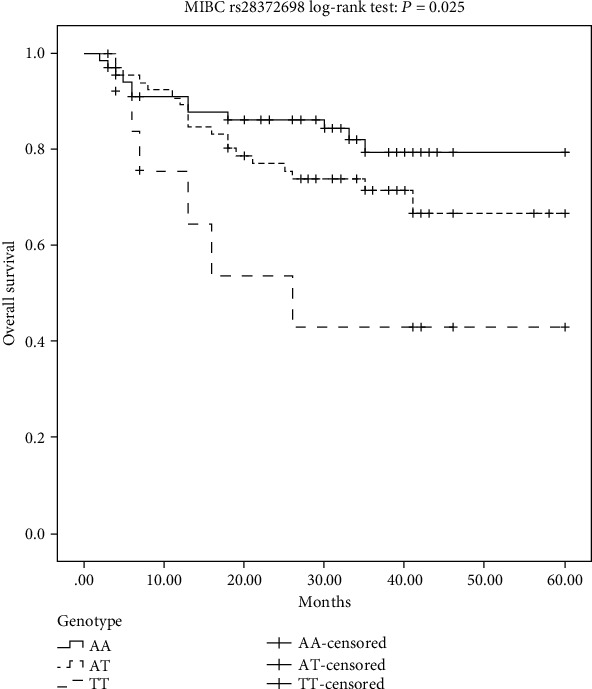
Kaplan-Meier overall survival curves for all of the analyzed MIBC patients categorized by *IL-32* rs28372698 in the codominant genetic models.

Kaplan-Meier plots indicated a significantly worse prognosis of MIBC patients carrying the TT homozygous genotype of *IL-32* rs28372698 compared to that of AA or AT genotypes (log-rank test: *P* = 0.015, Figure 1; *P* = 0.025, Figure 2). Furthermore, as shown in Table 4, the multivariate survival analyses reiterated that the TT genotype carriers (*P* = 0.028, OR (95%CI) = 2.77 (1.11-6.90)) had a worse overall survival rate in MIBC patients after adjustment for age, sex, and smoking status. However, no significant relationship was detected between the overall survival rate and another SNP (rs12934561) or between these two SNPs and the recurrence-free survival rate.

## 4. Discussion

Proinflammatory cytokines, such as tumor necrosis factor *α* (TNF-*α*), interleukin-8 (IL-8), interleukin-6 (IL-6), and interleukin-1*β* (IL-1*β*), could be induced by IL-32, which is often associated with inflammatory and oncogenic diseases (7, 32–34). However, no homologous relationship has been found between the structural basis of IL-32 and the known cytokines, and no extracellular signaling receptor of IL-32 1has been detected until now (7, 35, 36). IL-32 has nine splice variants, and all of the isoforms present differences in secondary structures, which lead to the variant tertiary protein structure and protein function (37).

Substantial reports have shown that IL-32 has different roles in various situations and pathways. Mabilleau and Sabokbar reported that IL-32 is capable of inducing a strong activation of ERK1/2 and Akt signaling and stimulating the release of interleukin-4 (IL-4) and interferon-*γ* (IFN-*γ*) in osteoclast formation and activation (38). The transcriptional coactivator p300 (EP300) and death-associated protein kinase-1 (DAPK-1) were found to occupy the inflammatory network nodes of IL-32, which affect both TNF-receptor 1-dependent and TNF-receptor 1-independent pathways (39). Yousif et al. demonstrated that IL-32 is associated with NF-*κ*B and p38 MAPK pathways in esophageal tumors in vivo (19), whereas in vitro, Oh et al. found that it is involved in the NF-*κ*B-STAT3 signaling pathway in colon cancer cells (40). Park et al. suggested that IL-32*β* could increase the invasion and migration of breast cancer through the EGFR-STAT3 pathway (26).

Several studies on the SNP of *IL-32* in cancer have been reported in recent years, and *IL-32* has even been linked to the patient outcome in some cancers. Our data revealed that the CC genotype of rs12934561 in *IL-32* was associated with an increased risk of BC, which is consistent with findings of previous studies in which the CC genotype was shown to relate closely with an increased susceptibility in lung cancer and endometrial cancer (16, 27). Moreover, Wang et al. showed that lung squamous carcinoma patients with the TT genotype of rs12934561 present a relatively poor survival rate compared with that of other patients (16). However, in our study, we demonstrated that the TC heterozygotes of rs12934561 are associated with a decreased risk of BC, which might be caused by the variant effects and functional pathways of nine isoforms in different organizations.

The rs28372698 T/A genetic variants were located on the 5′-UTR in the promoter region of *IL-32*. Plantinga et al. investigated a cohort of 139 TC patients and 138 healthy controls who carried the rs28372698 T/A genetic variants, revealing an increased risk of TC in patients with genetic variants of IL-32. Those patients required higher doses of cumulative radioactive iodine (RAI) to achieve successful tumor remission (14). Gonzalez-Hormazabal et al. used a combined attribute network implemented in multifactor dimensionality reduction software to analyze the gene-gene interactions between *IL-8*-251 A>T and *IL-32* rs28372698 T/A, and their results showed that the homozygote for both *IL-8*-251 T and *IL-32* rs28372698 T alleles presents a 2.63-fold risk in the developing gastric cancer (29). Furthermore, in moderate and well-differentiated lung cancer, the T allele of rs28372698 is associated with a poor prognosis (16), which is consistent with our data. In our study, the TT genotype of rs28372698 in *IL-32* was associated with a lower overall survival rate of MIBC patients, which indicated that IL-32 might be a potential biomarker for the prognosis of BC.

## 5. Conclusions

In conclusion, to the best of our knowledge, this is the first study to demonstrate the relationship between IL-32 and BC. The results indicate that SNP rs12934561 may be a potential risk factor for BC processes, and SNP rs28372698 is a significant forecast factor for BC prognosis. Nevertheless, our study has some limitations in terms of sample size and in the absence of the expression level of IL-32 in participants. The types and frequencies of genetic polymorphisms in variant ethnic populations differ, whereas only a cohort of southwest China was genotyped in this study. Thus, further studies in different populations and with larger sample sizes are required to reveal the potential function and mechanism of IL-32 in BC and to confirm these findings.

## Figures and Tables

**Table 1:** Characteristics of the study population.

Characteristics	NMIBC group	MIBC group	Controls
Sample size	170	151	437
Sex			
Male	131 (77.1%)	122 (80.8%)	336 (76.9%)
Female	39 (22.9%)	29 (19.2%)	101 (23.1%)
Age at first diagnosis (mean ± SD)	62.14 ± 12.87	65.70 ± 11.06	63.86 ± 6.94
Smoking status			
Smokers	85 (50.0%)	82 (54.3%)	199 (45.5%)
Nonsmokers	85 (50.0%)	69 (45.7%)	238 (54.5%)
Clinical stage			
Ta	10 (5.9%)	—	—
T1	160 (94.1%)	—	—
T2	—	89 (58.9%)	—
T3a	—	34 (22.5%)	—
T3b	—	17 (11.3%)	—
T4	—	11 (7.3%)	—
Tumor grade			
Low grade	114 (67.1%)	23 (15.2%)	—
High grade	56 (32.9%)	128 (84.8%)	—

**Table 2:** Primer sequences for genotyping two SNPs in the IL-32 gene.

SNP ID	Primer sequence	Restriction enzyme	Allele (bp)
rs12934561	F: 5′-GGCCTCACTCCTCACACAGT-3′	Hpy188III	C (20 + 155)
	R: 5′-CCCACAGGTGTTGGTTTCC-3′		T (175)
rs28372698	F: 5′-GTCAGAAGGACCTGGTCAGC-3′	Hpy188III	A (115)
	R: 5′-GTTGGAGGGGTGGCTAGTC-3′		T (21 + 94)

**Table 3:** Distribution of SNPs in *IL-32* among patients and controls and their association with bladder cancer risk.

		rs28372698				rs12934561				
Model	Genotype	Patients *N* (%)	Controls *N* (%)	OR (95% CI)	*P*	Genotype	Patients *N* (%)	Controls *N* (%)	OR (95% CI)	*P*
Codominant	AA	144 (44.9%)	215 (49.2%)	1.00 (reference)		TT	127 (39.6%)	151 (34.5%)	1.00 (reference)	
	AT	147 (45.8%)	193 (44.2%)	1.15 (0.85-1.54)	0.24	TC	115 (35.8%)	207 (47.4%)	**0.65 (0.47-0.90)**	**0.004**
	TT	30 (9.3%)	29 (6.6%)	1.59 (0.91-2.78)		CC	79 (24.6%)	79 (18.1%)	1.18 (0.79-1.75)
Dominant	AA	144 (44.9%)	215 (49.2%)	1.00 (reference)		TT	127 (39.6%)	151 (34.5%)	1.00 (reference)	
	AT/TT	177 (55.1%)	222 (50.8%)	1.20 (0.90-1.61)	0.21	TC/CC	194 (60.4%)	286 (65.5%)	0.79 (0.59-1.08)	0.14
Recessive	AA/AT	291 (90.7%)	408 (93.4%)	1.00 (reference)		TT/TC	242 (75.4%)	358 (81.9%)	1.00 (reference)	
	TT	30 (9.3%)	29 (6.6%)	1.47 (0.86-2.50)	0.15	CC	79 (24.6%)	79 (18.1%)	**1.47 (1.04-2.08)**	**0.03**
Overdominant	AA/TT	174 (54.2%)	244 (55.8%)	1.00 (reference)		TT/CC	206 (64.2%)	230 (52.6%)	1.00 (reference)	
	AT	147 (45.8%)	193 (44.2%)	1.08 (0.80-1.43)	0.64	TC	115 (35.8%)	207 (47.4%)	**0.61 (0.45-0.82)**	**0.001**
	Allele									
	A	435 (67.8)	623 (71.3)	1.18 (0.95-1.47)	0.14	T	369 (57.5)	509 (58.2)	1.03 (0.84-1.27)	0.77
	T	207 (32.2)	251 (28.7)			C	273 (42.5)	365 (41.8)		

*N* corresponds to the number of individuals. Boldfaced values indicate a significant difference at the 5% level.

**Table 4:** Association between SNPs in IL-32 and patient outcome.

SNP/genotype	NMIBC						MIBC					
	Alive/dead, *N*	HR (95% CI)^a^	*P*	Recurrence/nonrecurrence	HR (95% CI)^a^	*P*	Alive/dead, *N*	HR (95% CI)^a^	*P*	Recurrence/nonrecurrence	HR (95% CI)^a^	*P*
rs28372698												
AA	68/7			52/23			57/12			50/19		
AT	72/6			59/19			50/19			46/23		
TT	17/0			11/6			7/6			8/5		
Dominant		0.66 (0.22-1.98)	0.46		0.72 (0.40-1.28)	0.26		1.83 (0.92-3.65)	0.09		1.54 (0.85-2.78)	0.16
Recessive		NA	0.98		1.57 (0.66-3.76)	0.31		**2.77 (1.11-6.90)**	**0.028**		2.06 (0.79-5.36)	0.14
Overdominant		0.86 (0.29-2.59)	0.79		0.60 (0.33-1.09)	0.09		1.22 (0.64-2.32)	0.56		1.24 (0.70-2.21)	0.46
rs12934561												
TT	64/5			46/23			45/13			40/18		
TC	61/3			47/17			38/13			39/12		
CC	32/5			29/8			31/11			25/17		
Dominant		0.92 (0.29-2.95)	0.89		0.70 (0.39-1.26)	0.23		1.24 (0.61-2.53)	0.55		0.91 (0.49-1.68)	0.76
Recessive		1.92 (0.61-6.08)	0.27		0.57 (0.27-1.24)	0.16		0.95 (0.46-1.93)	0.88		1.46 (0.80-2.68)	0.22
Overdominant		0.47 (0.13-1.76)	0.27		1.03 (0.56-1.89)	0.92		1.32 (0.66-2.67)	0.43		0.60 (0.31-1.18)	0.14

*N* corresponds to the number of individuals. ^a^Adjusted by age, sex, and smoking status. Boldfaced values indicate a significant difference at the 5% level.

## Data Availability

The data used to support the findings of this study are currently under embargo while the research findings are commercialized. Requests for data, 6 months after publication of this article, will be considered by the corresponding authors.

## Supplementary Materials

Supplementary Materials Supplementary Table 1: association between SNPs in *IL-32* and patient's characteristics.
