# Supplementary material for: Associations between Interleukin-32 Gene Polymorphisms rs12934561 and rs28372698 and Susceptibilities to Bladder Cancer and the Prognosis in Chinese Han Population
Source: Dis Markers. 2020 Nov 5;2020:8860445. doi: 10.1155/2020/8860445 (PMC7661138; doi:10.1155/2020/8860445)
Supplement: Supplementary Materials — Supplementary Table 1: association between SNPs in IL-32 and patient's characteristics. [file 8860445.f1.docx]

**Supplementary Table 1.** Association between SNPs in *IL-32* and patient’s characteristics.

| Characteristics | rs28372698 | | | | | |  | rs12934561 | | | | | |
| --- | --- | --- | --- | --- | --- | --- | --- | --- | --- | --- | --- | --- | --- |
|  | Genotype | | | OR (95% CI) **^a^** | | |  | Genotype | | | OR (95% CI) **^a^** | | |
|  | AA | AT | TT | Dominant (AA *VS.* AT/TT) | Recessive (AA/AT *VS.* TT) | Overdominant (AA/TT *VS.* AT) |  | TT | TC | CC | Dominant (TT *VS.* TC/CC) | Recessive (TT/TC *VS*. CC) | Overdominant (TT/CC *VS.* TC) |
| **Age** | | | | | | | | | | | | | |
| ≤64 | 72 (42.1%) | 81 (47.4%) | 18 (10.5%) | 1.25 (0.80-1.96) | 1.31 (0.60-2.88) | 1.14 (0.73-1.79) |  | 61 (35.7%) | 69 (40.4%) | 41 (24.0%) | 1.41 (0.89-2.23) | 0.97 (0.58-1.63) | 1.47 (0.91-2.35) |
| ＞64 | 72 (48.0%) | 66 (44.0%) | 12 (8.0%) |  |  |  |  | 66 (44.0%) | 46 (30.7%) | 38 (25.3%) |  |  |  |
| **Sex** | | | | | | | | | | | | | |
| Male | 112 (44.3%) | 115 (45.5%) | 26 (10.3%) | 1.09 (0.59-1.99) | 1.86 (0.57-6.08) | 0.91 (0.49-1.66) |  | 101 (39.9%) | 92 (36.4%) | 60 (23.7%) | 0.76 (0.41-1.42) | 0.80 (0.41-1.59) | 0.90 (0.47-1.72) |
| Female | 32 (47.1%) | 32 (47.1%) | 4 (5.9%) |  |  |  |  | 26 (38.2%) | 23 (33.8%) | 19 (27.9%) |  |  |  |
| **Smoking Status** | | | | | | | | | | | | | |
| Smokers | 73 (43.7%) | 77 (46.1%) | 17 (10.2%) | 1.03 (0.63-1.70) | 0.96 (0.41-2.22) | 1.05 (0.64-1.73) |  | 60 (35.9%) | 67 (40.1%) | 40 (23.9%) | 1.43 (0.86-2.38) | 1.00 (0.56-1.80) | 1.45 (0.86-2.46) |
| Non-smokers | 71 (46.1%) | 70 (45.5%) | 13 (8.4%) |  |  |  |  | 67 (43.5%) | 48 (31.2%) | 39 (25.3%) |  |  |  |
| **Tumor grade** | | | | | | | | | | | | | |
| Low-grade | 60 (43.8%) | 63 (46.0%) | 14 (10.2%) | 1.04 (0.62-1.76) | 1.11 (0.45-2.71) | 1.01 (0.60-1.70) |  | 59 (43.1%) | 47 (34.3%) | 31 (22.6%) | 0.74 (0.43-1.27) | 0.98 (0.53-1.81) | 0.75 (0.43-1.30) |
| High-grade | 84 (45.6%) | 84 (45.6%) | 16 (8.7%) |  |  |  |  | 68 (37.0%) | 68 (37.0%) | 48 (26.1%) |  |  |  |
| **Tumor stage** | | | | | | | | | | | | | |
| Ta-T1 | 75 (44.1%) | 78 (45.9%) | 17 (10.0%) | 1.03 (0.61-1.72) | 1.10 (0.45-2.69) | 0.99 (0.59-1.67) |  | 69 (40.6%) | 64 (37.6%) | 37 (21.8%) | 1.03 (0.61-1.76) | 0.73 (0.40-1.33) | 1.34 (0.78-2.32) |
| T2-T4 | 69 (45.7%) | 69 (45.7%) | 13 (8.6%) |  |  |  |  | 58 (38.4%) | 51 (33.8%) | 42 (27.8%) |  |  |  |

*N* corresponds to the number of individuals.

^a^ Adjusted by age, sex, smoking status, tumor grade and tumor stage.
